# Specific alterations in riboproteomes composition of isonicotinic acid treated arabidopsis seedlings

**DOI:** 10.1007/s11103-022-01332-2

**Published:** 2023-02-15

**Authors:** Zainab Fakih, Mélodie B. Plourde, Charlène Eugénie Tomi Nkouankou, Victor Fourcassié, Sylvie Bourassa, Arnaud Droit, Hugo Germain

**Affiliations:** 1 Department of Chemistry, Biochemistry and Physics and Groupe de Recherche en Biologie Végétale, Université du Québec à Trois-Rivières, G9A 5H9 Trois-Rivières, Québec Canada; 2 Proteomics Platform, Centre de recherche du CHU de Québec, Faculty of Medicine, Université Laval, G1V 4G2 Québec City, Québec Canada

**Keywords:** *Arabidopsis thaliana*, Translation regulation, Proteomics, Ribosomal protein small subunit (RPS), Ribosomal protein large subunit (RPL), Plant immunity

## Abstract

Plants have developed strategies to deal with the great variety of challenges they are exposed to. Among them, common targets are the regulation of transcription and translation to finely modulate protein levels during both biotic and abiotic stresses. Increasing evidence suggests that ribosomes are highly adaptable modular supramolecular structures which remodel to adapt to stresses. Each *Arabidopsis thaliana* ribosome consists of approximately 81 distinct ribosomal proteins (RPs), each of which is encoded by two to seven genes. To investigate the identity of ribosomal proteins of the small subunit (RPS) and of the large subunit (RPL) as well as ribosomes-associated proteins, we analysed by LC/MS/MS immunopurified ribosomes from *A. thaliana* leaves treated with isonicotinic acid (INA), an inducer of plant innate immunity. We quantified a total of 2084 proteins. 165 ribosome-associated proteins showed increased abundance while 52 were less abundant. Of the 52 identified RPS (from a possibility of 104 encoding genes), 15 were deregulated. Similarly, from the 148 possible RPL, 80 were detected and 9 were deregulated. Our results revealed potential candidates involved in innate immunity that could be interesting targets for functional genomic studies.

**Supplementary information:**

The online version contains supplementary material available at 10.1007/s11103-022-01332-2.

## Introduction

Plants are exposed to a variety of abiotic and biotic stresses which induce numerous adverse effects and eventually reduce growth, development, and overall productivity (Pandey et al. 2017; Verma et al. 2013). Depending on the external stimuli, pathogen lifestyles, and infection strategies, plants have developed multiple defense tactics to respond to these adverse conditions, leading to a massive reprogramming of the cell. Studies on pathogen-triggered immunity (PTI) and effector-triggered immunity (ETI) have shown that significant transcriptome and proteome changes occur during plant defense (Jones et al. 2004, 2006). Various factors can affect the correlation between mRNA transcripts and proteins levels such as different half-lives of mRNAs and proteins, mRNA structural features e.g. 5’cap m^7^GpppN, translation enhancer in the poly(A), internal ribosomal entry sites (IRESs) promoting cap-independent translation, hairpins and upstream open reading frames (uORFs) affecting translation of the main open reading frame, protein–RNA interactions and ribosome occupancy (Fütterer and Hohn 1996; Haider and Pal 2013; Merchante et al. 2017; Schwanhäusser et al. 2011).

Previously viewed as a passive mediator catalyzing protein synthesis from messenger RNA, ribosomes are now considered to be dynamic macromolecular complexes with specialized roles in the cell (Genuth and Barna 2018). *Arabidopsis thaliana* ribosomes are composed of the 40S subunit, consisting of the 18S rRNA and 33 ribosomal proteins (RPs), while the 60S subunit contains three rRNA (25S, 5.8S, and 5S) and 48 RPs. Those 81 distinct RPs are encoded by a total of 252 genes; thus, each RP is encoded by two to seven paralogous genes, with an average of three (Barakat et al. 2001; Carroll et al. 2008). Most RP genes are expressed preferentially at different developmental points, in different cell types, or under different conditions (Carroll et al. 2008). This differential expression between gene families as well as within specific ribosomal gene families points to ribosome heterogeneity, which includes the absence of specific RPs from the canonical ribosome structure, RP paralogs exchange, rRNAs sequence variation and posttranscriptional modifications, RPs posttranslational modifications, and possibly additional variations of the ribosome-associated proteome (Browning and Bailey-Serres 2015). Furthermore, different ribosome types could preferentially translate specific subsets of mRNAs and thus regulate protein synthesis under particular cell conditions (Giavalisco et al. 2005; Li and Wang 2020). Functionally specialized ribosomes would appear after a specific cue to shape an acclimated proteome (Ferretti and Karbstein 2019; Genuth and Barna 2018; Martinez-Seidel et al. 2020a, b).

The objective of the present study is to provide insights into the riboproteome when the plant defense response to biotic stress is induced by isonicotinic acid (INA), an analog of salicylic acid (SA). Using label-free relative quantitation proteomics, we quantified *A. thaliana* ribosomal and ribosome-associated proteins and identified 217 differentially expressed proteins. Out of the 252 ribosomal proteins annotated in TAIR10, 24 were differentially accumulated in *Arabidopsis* leaves following INA treatment. The 5’ upstream region of all RP genes was also assessed *in silico* for the presence of cis elements. Our results demonstrate the ability of this approach to address the dynamic nature of the riboproteome and suggest that specialized ribosomes or certain ribosomal proteins might be required during the defense response.

## Materials and methods

### Plant growth conditions and INA treatment

Transgenic *A. thaliana* plants expressing FLAG-RPL18 were obtained from Professor Peter Moffett (Université de Sherbrooke, Qc, Canada). The seeds were grown in soil (AgroMix), in a growth chamber after a stratification period of 48 h at 4 ºC. The plant growth chamber was maintained at 22 ºC, 60% relative humidity, and with a 14 h/10 h light/dark cycle. INA was used to induce plant defense as it was shown to induce a response similar to salicylic acid and pathogen infection (Conrath et al. 1995). 4-week-old plants were sprayed to imminent runoff with an aqueous solution of 0.65 mM INA containing 0,05% Sylgard 309 surfactant, whereas mock treatment consisted of only the Sylgard 309 aqueous solution, leaf tissues were harvested 24 h after being sprayed with INA as previously described (Cheng et al. 2009).

### Gene expression analysis

Gene expression analysis was performed on RNA extracted from 4-week-old soil-grown plants using the Genezol Total RNA kit (Geneaid) according to the manufacturer’s instructions. RNA quality was assessed by agarose gel electrophoresis and quantified by spectrophotometry. 1 µg of each sample was reverse transcribed into cDNA with the M-MuLV Reverse Transcriptase (New England Biolabs Canada). Quantitative RT-PCR amplification was done on a CFX Connect detection system (Bio-Rad Laboratories, Mississauga, On, CA) using SYBR Green PCR Master Mix (Bioline). 100 ng cDNA template and 0.4 µM of each primer (listed in Supplementary Table 1) were used in a final volume of 20 µl. The qRT-PCR thermal profile was 95 ˚C for 2 min, 40 cycles of 95 ˚C for 5 s, 60 ˚C for 10 s, and 72 ˚C for 5 s. The data were analyzed with CFX Maestro qPCR software. *At1g13320* was used as a reference gene since it was previously demonstrated to be amongst the most stable genes in Arabidopsis (Czechowski et al. 2005) and was also used as a reference gene in a similar study (Vos et al. 2015). The expression level of each gene was calculated according to the ^ΔΔ^Ct method. Three technical replicates for each treatment were analyzed.

### Ribosome immunopurification

Leaves from three independent batches of transgenic FLAG-RPL18B *A. thaliana* plants were sprayed with INA and collected after 24 h for ribosome purification. Ribosomes were isolated using the previously published protocol (Zanetti et al. 2005) with minor modifications. Frozen, pulverized leaf tissue (~ 2.5 g) was mixed with two volume of polysome extraction buffer [(PEB); 200 mM Tris-HCl (pH 9.0), 200 mM KCl, 36 mM MgCl_2_, 10 mM EGTA, 1 mg/ml heparin, 1 mM DTT, 50 µg/ml cycloheximide, 50 µg/ml chloramphenicol, 2% (v/v) Triton X-100, 2% (v/v) Tween 40, 2% (w/v) Brij-35, 2% (v/v) NP-40, 2% (v/v) polyoxyethylene (10) tridecyl ether and 1% (w/v) sodium deoxycholate] and incubated for 30 min at 4 °C with gentle rotation. Homogenates were clarified by two consecutive centrifugations at 16,000xg for 10 min at 4 °C. The supernatants were incubated with 100 µl (packed volume) of buffer-equilibrated anti-FLAG M2 magnetic beads (Sigma-Aldrich, USA) for 2 h at 4 °C with gentle agitation. The beads were washed three times with 1 ml of wash buffer (100 mM Tris–HCl pH 8.5, 200 mM KCl, 25 mM EGTA pH 8.0, 36 mM MgCl_2_) at 4 °C, then five times with 50 mM ammonium bicarbonate buffer and stored at -80 °C. The beads were sent on dry ice to the Proteomics platform of the Centre hospitalier universitaire de Québec, where they were further processed.

### Sample preparation for mass spectrometry

Proteins were on-beads digested using 0.1 µg of modified porcine trypsin (sequencing grade, Promega, Madison, WI) in 50 mM ammonium bicarbonate for 5 h at 37 °C. Digestion was stopped with 5% formic acid (FA) and peptides were eluted from the beads with 60% acetonitrile (ACN) + 0.1% FA. The tryptic peptides were desalted on a C18 stage tip, lyophilized, re-dissolved in 10 µl LC loading solvent and peptides quantities were estimated with 205 nm absorbance (Nanodrop, Thermo Scientific). Peptide samples (1 µg) were injected and separated using a Dionex UltiMate 3000 nanoRSLC chromatography system (Thermo Fisher Scientific) connected to an Orbitrap Fusion mass spectrometer (Thermo Fisher Scientific, San Jose, CA, USA) equipped with a nanoelectrospray ion source. Peptides were trapped at 20 µl/min in loading solvent (2% acetonitrile, 0.05% TFA) on a 5 mm x 300 μm C18 PepMap cartridge (Thermo Fisher Scientific) for 5 min. Then, the pre-column was switched online with a 50 cm x 75 μm internal diameter separation column (PepMap Acclaim column, ThermoFisher) and the peptides were eluted with a linear gradient from 5 to 40% solvent B (A: 0.1% formic acid, B: 80% acetonitrile, 0.1% formic acid) for 90 min at 300 nl/min. Mass spectra were acquired using a data dependent acquisition mode using Thermo XCalibur software version 4.1.50. Full scan mass spectra (350 to 1800 m/z) were acquired in the orbitrap using an AGC target of 4e5, a maximum injection time of 50 ms and a resolution of 120 000. Internal calibration using lock mass on the m/z 445.12003 siloxane ion was used. Each MS scan was followed by acquisition of fragmentation MSMS spectra of the most intense ions for a total cycle time of 3 s (top speed mode). The selected ions were isolated using the quadrupole analyzer with 1.6 m/z windows and fragmented by Higher energy Collision-induced Dissociation (HCD) with 35% of collision energy. The resulting fragments were detected by the linear ion trap in rapid scan rate with an AGC target of 1e4 and a maximum injection time of 50 ms. Dynamic exclusion of previously fragmented peptides was set for a period of 30 s and a tolerance of 10 ppm.

### Protein identification and data analysis

Mass spectra were searched against the *A. thaliana* protein sequence database (Uniprot Arabidopsis thaliana UP000006548 version of August 24, 2020) using the search engine Andromeda integrated into the MaxQuant software (version 1.6.10.43) assuming the digestion enzyme trypsin. Carbamidomethyl cysteine and methionine oxidation or acetylation were set as fixed and variable modifications, respectively. For protein validation, a false discovery rate (FDR) of 1% was allowed at peptide and protein level based on a target/decoy search. Text files generated by MaxQuant were analysed using the R software (version 4.0.4). For data processing, only MaxQuant normalized LFQ intensities from the proteinGroups.txt file were considered. Decoy proteins and potential contaminants were excluded from the analysis. For each sample, a noise value corresponding to the 0.01 percentile of all LFQ intensities of said sample was calculated. This noise value was imputed when an intensity value was missing from a sample. Only proteins which presented intensity values (not noise imputed value) in 100% of the replicates of one group were considered as quantifiable proteins and kept for further analysis. Among these, only proteins identified with at least two razor unique peptides were kept for analysis. Outputs from individual runs (2 treatments, 6 purifications) were merged and filtered in Excel and multiple hits were removed to obtain the number of distinct proteins. The average LFQ intensity value from each experimental condition was calculated for each protein, then the values of the INA treated samples were divided by those of control samples. The Limma statistical test was performed to determine the probability value (*P*-value) and the Benjamini Hochberg adjusted probability value (*q-*value) of variation for each protein. Significant change between treated and untreated plants was conservatively defined as absolute log_2_ value of fold change ≥ 1.5 and *q-*value < 0.05 as previously described and with at least 2 unique peptides. Proteins were divided into ribosomal proteins and potential ribosome-associated proteins based on the annotations, and RPs were further subdivided into RPS and RPL proteins. Mass spectrometry data and relative quantitation results are publicly available on the MassiVE repository (https://massive.ucsd.edu/ProteoSAFe/static/massive.jsp) with the identifier MSV000089714.

### Visualization of RP localization

The RP localization was visualized using PyMol software (http://www.pymol.org). To the best of our knowledge, the wheat structure is the currently most complete and adequate, high-resolution plant cytosolic ribosome structure in the PDB database and represents the current canonical structure model of plant 80S ribosomes. Given that a high-resolution structure of the mature, translating Arabidopsis cytosolic ribosome has yet to be made publically available, the Triticum aestivum 80S ribosomal structure published by Armache et al. 2010b was used as reference for our visualizations (PDB ID 4v7e). Using protein BLAST comparisons, Martinez-Seidel et., 2020 verified the RP identity of Arabidopsis RP.

Homologs of the protein entries linked to the macromolecular Crystallographic Information Files (mmCIF) of the wheat 80S structure model. The Arabidopsis RPs are adequately matched to the wheat RPs mapped in the 80S structure model.

### 5’ upstream region analysis

Since 24 ribosomal proteins (RP) exhibited differential expression following INA treatment, we checked for the presence of putative cis elements in the 5’ upstream region of the RP genes using an *in silico* approach. We considered only the 5’ upstream region and did not include introns to compare more homogenous data since several ribosomal gene are intronless (Supplementary Table 2). The nucleotide sequence of the 5’ upstream region of each of the RPS genes was submitted to PlantCARE (Cis-Acting Regulatory Elements) Database10 (Dhadi et al. 2009; Ding et al. 2011) to identify regulatory elements. We also compared the 5’ upstream region of some of the significant deregulated RP genes in other databases, such as PlantPAN 3.0, and found functional similarity with the PlantCare database.

## Results

### Ribosome enrichment following immunity activation

**Fig. 1:**
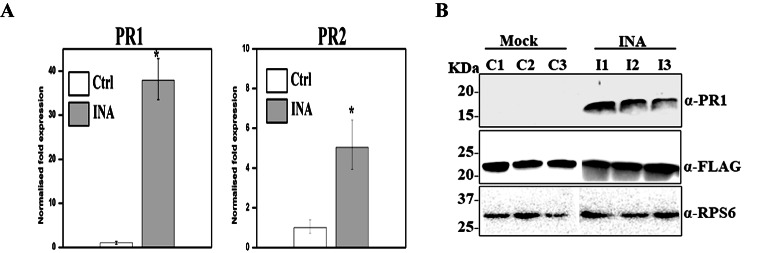
Ribosome enrichment following immunity activation (A) qRT-PCR analysis of INA-induced *PR1* and *PR2* expression. *At1G13320* was used as the reference gene. The error bars represent ± SD (n = 3). The asterisks represent a significant difference between treated and mock-treated samples. *P-values < 0.01, Student’s t-test. (B) Western blot analysis of ribosome enriched protein extracts of untreated and INA-treated leaf tissues of the FLAG-RPL18 transgenic *A. thaliana* line. Top panel: PR1 accumulation in INA samples, middle panel: FLAG-RPL18, bottom panels: detection of the small subunit protein RPS6

To assess the efficiency of SA defense pathway induction, we quantified the mRNA levels of two reporter genes, *PR1* and *PR2* (pathogenesis related protein). It has been shown that exogenous application of SA, or of one of its functional analogs (2,6-dichloroisonicotinic acid [INA] can activate *PR* gene expression and resistance in plants without pathogen inoculation. PRs possess antimicrobial activity and are thought to contribute to the broad-spectrum resistance (Ali et al. 2018; Chandrashekar et al. 2018). 24 h post-INA treatment, both *PR1* and *PR2* were upregulated by 37.9- and 5-fold respectively (Fig. 1 A). We also detected PR1 protein accumulation in extracts of INA treated tissues but not in untreated leaves (Fig. 1B). The affinity purification of ribosomes was performed using FLAG-tagged large ribosomal protein RPL18, which has successfully been used in studies of the Arabidopsis translatome and proteome (Eskelin et al. 2019; Hummel et al. 2012; Meteignier et al. 2017; Zanetti et al. 2005) and RPL18-FLAG level was assessed prior to immunoprecipitation (Fig. 1B. The presence of small ribosomal proteins was verified by western blotting of the small subunit ribosomal protein RPS6 in the mock and treated samples (Fig. 1B).

### The riboproteome is deregulated by INA treatment

A total of 2813 proteins were detected in the ribosome preparations from INA treated and control plants (Figure S1). 2376 proteins were detected in all biological replicates of at least one experimental condition and were thus considered as quantifiable proteins. Among these, 2084 proteins were identified with at least two razor unique peptides and kept for the analysis (Figure S1; Table S3). The presence of non-ribosomal proteins was examined in the combined data of all samples, revealing 1882 distinct non-ribosomal proteins identified on the basis of unique peptides (Table S4). Many non-ribosomal proteins important for translation regulation, such as elongation factors (At1g30230, At3g18760), ribosome assembly factors (At1g25260) and ribosome biogenesis proteins (AT1G52930) were found to associate with ribosomes.

**Fig. 2:**
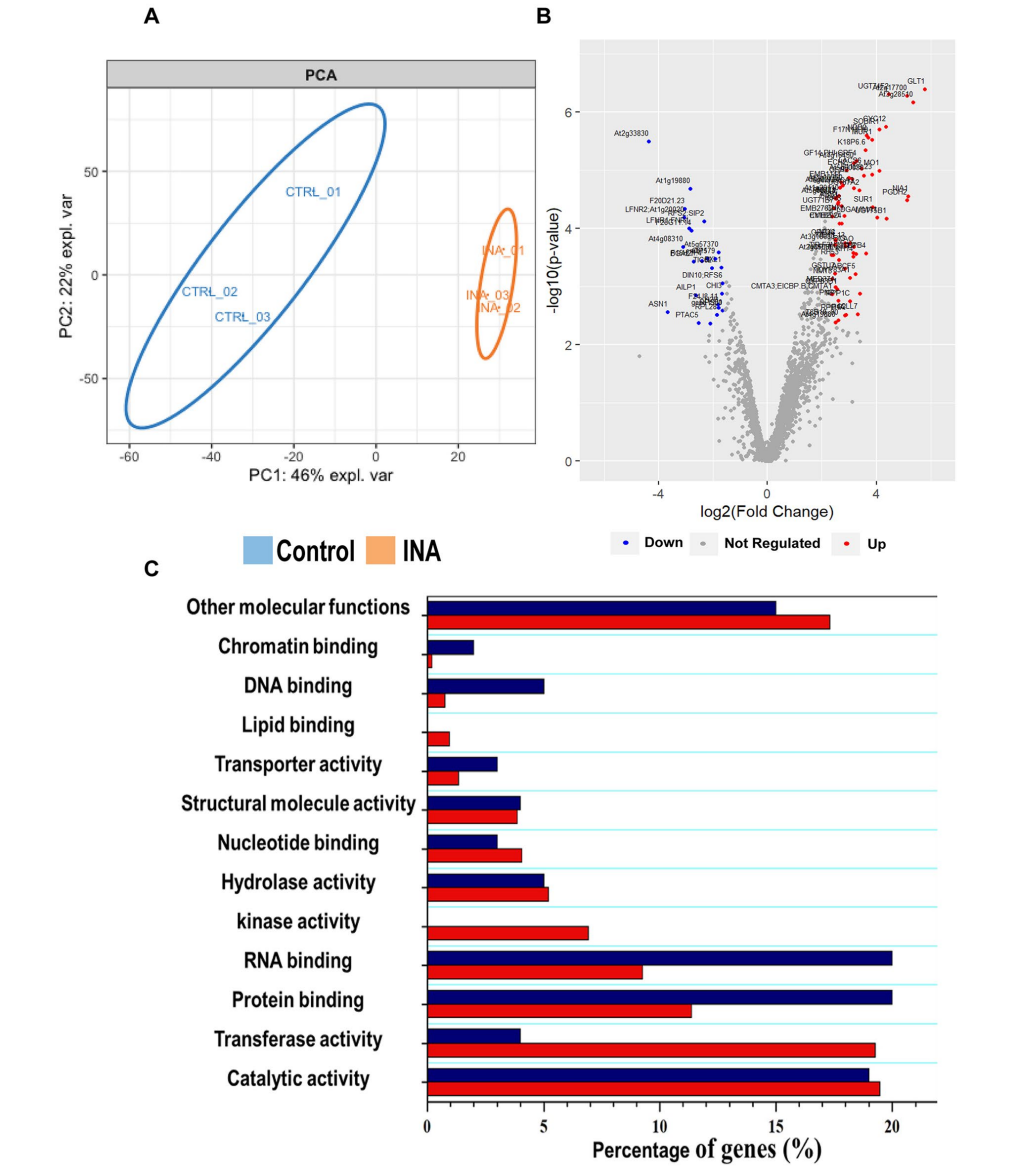
The riboproteome is deregulated by INA treatment. (A) PCA of all the mass spectra matched peaks obtained from immunopurified ribosomal preparations of INA treated and control leaves. Ellipses encircle biological replicates. (B) Volcano plot of the deregulated proteins. Significant deregulation was set as an absolute log_2_ value of fold change ≥ 1.5 and a *q-*values < 0.05. (C) GO functional analysis of the deregulated proteins, retrieved using the PANTHER Classification System

To globally present the variation between the INA treated and control ribosomal preparations, a Principal Component Analysis (PCA) was performed using log_2_ transformed normalized protein intensities with noise-imputed missing values. In this plot (Fig. 2A), the INA-treated and control samples are clearly separated. The biological replicates from each treatment clustered together, indicating that the variance between the replicates is much smaller than between treatments.

We next sought to assess and quantify the dynamic changes occurring in the riboproteome as a consequence of plant immunity induction with INA. All proteins for which label-free relative quantitation (LFQ) data was available in both treated and control samples, and across all three biological replicates in one of the two conditions, were analysed to identify significant changes in protein abundance (Table S5). These analyses identified 165 proteins with increased abundance and 52 proteins with reduced levels in treated samples (Fig. 2B). Detailed differential abundance ratios are shown in supplementary Figure S2 and Table S5. To identify processes that may be perturbed during the induction of plant immunity, we examined the predicted functions of the affected proteins. Gene ontology (GO) terms associated with catalytic activity, hydrolase activity, nucleotide binding and structural molecule activity were highly ranked in proteins that both increased and decreased in abundance upon induction (Fig. 2C). In addition, proteins annotated with transferase activity and kinase activity were highly ranked in proteins which increased in abundance during treatment, while RNA binding and protein binding were among the top GO terms for downregulated proteins (Fig. 2C). These data allowed us to determine the average protein composition of the ribosome population (the 60S subunit and intact 80S ribosomes, as well as large polysomes) and its associated proteins and evaluate relative differences in the Arabidopsis riboproteome.

### Small subunit ribosomal protein levels change in response to INA treatment

We next examined the presence of the 40S small subunit proteins (RPS) in our ribosomal enriched samples, since the immunoprecipitation was performed with FLAG-tagged RPL18 we can safely assumed that detected RPS proteins were part of ribosome comprising the small and large ribosomal subunit. Notably, our approach identified 52 distinct RPS from 30 of the 33 families (Table 1; Table S5) and 50% of the 104 genes encoding RPS were identified in all replicates. Within the filtered LC-MS/MS data, we did not have hits for RPS12, RPS21 and RPS29 but one unique peptide for RPS21B, RPS21C and RPS29A were detected in the raw data (Table S3).

**Table 1:** RPS detected by MS analysis in this study

Family	AGI code	Name	MW (kD)	Razor unique peptides
***SA***	At1g72370	**RPSaA**	32.3	8
	At3g04770	RPSaB	30.7	4
***S2***	At1g58380	RPS2A	30.7	8
	Q93VB8	RPS2B	30.8	4
	At2g41840	**RPS2C**	30.9	12
***S3***	At2g31610	**RPS3A**	27.5	15
	At3g53870	RPS3B	27.3	2
	At5g35530	RPS3C	27.5	4
***S3a***	At3g04840	RPS3aA	29.9	22
	At4g34670	RPS3aB	29.8	8
***S4***	At2g17360	RPS4A	30.1	3
	At5g07090	RPS4B	29.9	16
**S5**	At2g37270	**RPS5A**	23.0	9
	At3g11940	RPS5B	29.9	6
***S6***	At4g31700	RPS6A	28.4	5
	At5g10360	RPS6B	28.1	13
***S7***	Q9C514	**RPS7A**	21.9	9
	At5g16130	RPS7C	22.1	12
***S8***	At5g20290	RPS8A	24.1	9
***S9***	At5g15200	RPS9B	23.0	13
	At5g39850	**RPS9C**	23.2	10
**S10**	At4g25740	RPS10A	19.4	3
	At5g52650	**RPS10C**	19.8	3
***S11***	At3g48930	RPS11A	18.0	3
	At5g23740	**RPS11C**	17.7	2
***S13***	At3g60770	RPS13A	17.0	11
***S14***	At2g36160	RPS14A	16.3	12
***S15***	At1g04270	**RPS15A**	17.1	8
	At5g09500	RPS15C	16.7	6
	At5g09510	RPS15D	17.1	2
***S15a***	At1g07770	RPS15aA	14.8	6
***S16***	At5g18380	RPS16C	16.6	9
***S17***	At5g04800	**RPS17D**	16.0	4
***S18***	At1g22780	RPS18A	17.5	7
***S19***	At5g15520	RPS19B	15.8	9
	At5g61170	**RPS19C**	15.7	9
***S20***	At3g45030	RPS20A	13.1	4
***S23***	At5g02960	**RPS23B**	16.2	6
***S24***	At3g04920	**RPS24A**	15.4	9
	At5g28060	RPS24B	15.4	3
***S25***	At2g21580	RPS25B	12.1	8
	At4g34670	RPS25D	12.1	2
	At4g39200	RPS25E	12.1	5
***S26***	At3g56340	RPS26C	14.6	5
***S27***	At3g61110	RPS27B	9.5	3
	At5g47930	**RPS27D**	9.5	2
***S27a***	At1g23410	RPS27aA	17.7	7
***S28***	At3g10090	RPS28A	7.4	2
***S30***	At2g19750	RPS30A	6.9	3
***RACK1***	At1g18080	RACK1A	35.7	10
	At1g48630	RACK1B	35.8	2
	At3g18130	**RACK1C**	35.8	5

**Fig. 3:**
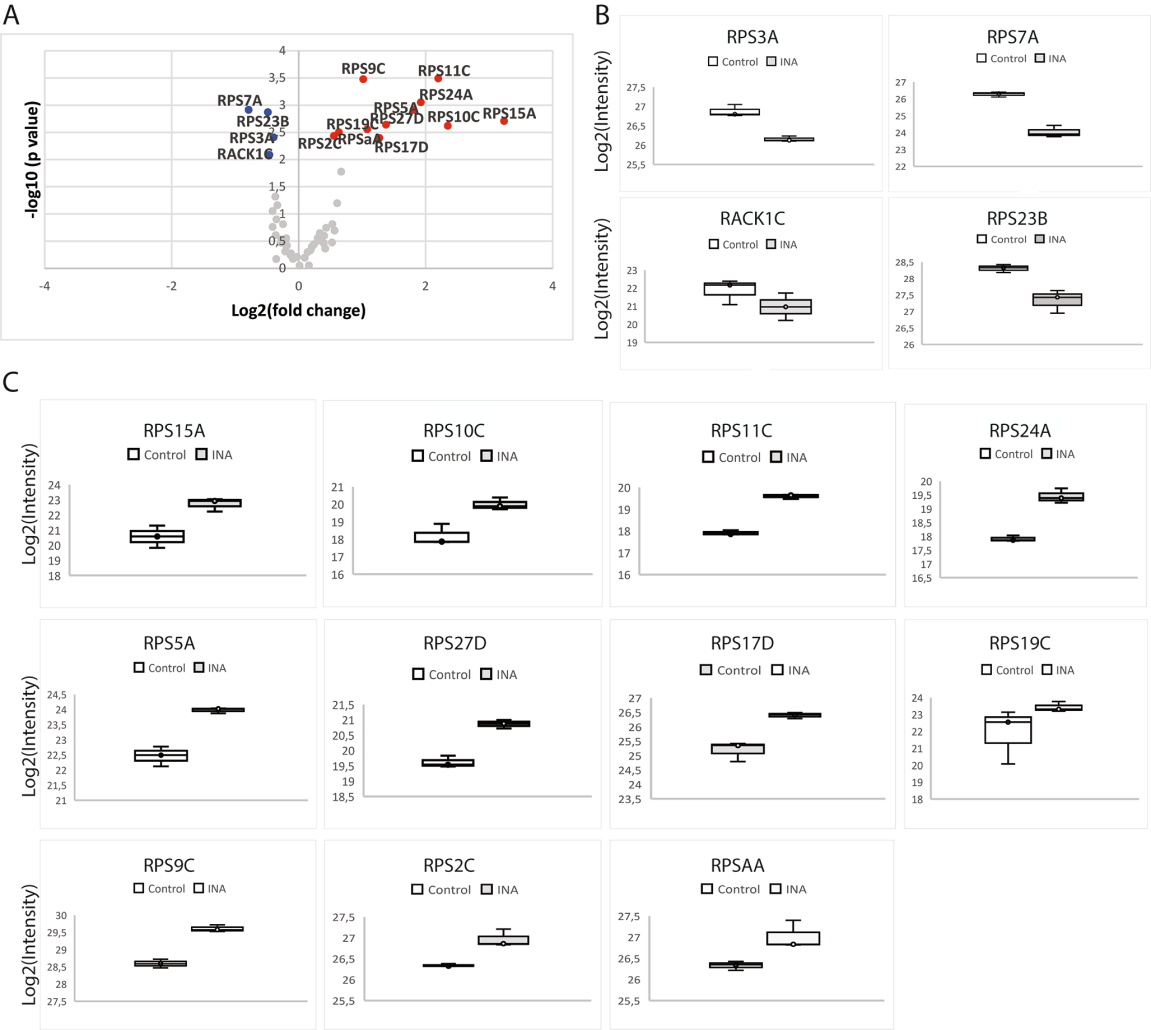
Small subunit ribosomal protein levels change in response to INA treatment (A) Volcano plots of deregulated RPS: downregulated in blue and upregulated in red. Significant deregulation was set as an absolute log_2_ value of fold change ≥ 1.5 and a *q-*values < 0.05 (B, C) Box plots showing the differences and replicate distribution of replicates for each statistically significant downregulated (B) and upregulated (C) proteins. The asterisks represent a significant difference between treated and mock-treated samples. *P-values < 0.05, **P-values < 0.01, limma t-test. (C) Heat map showing the ten most upregulated and downregulated RPS paralogs detected in this study

**Fig. 4:**
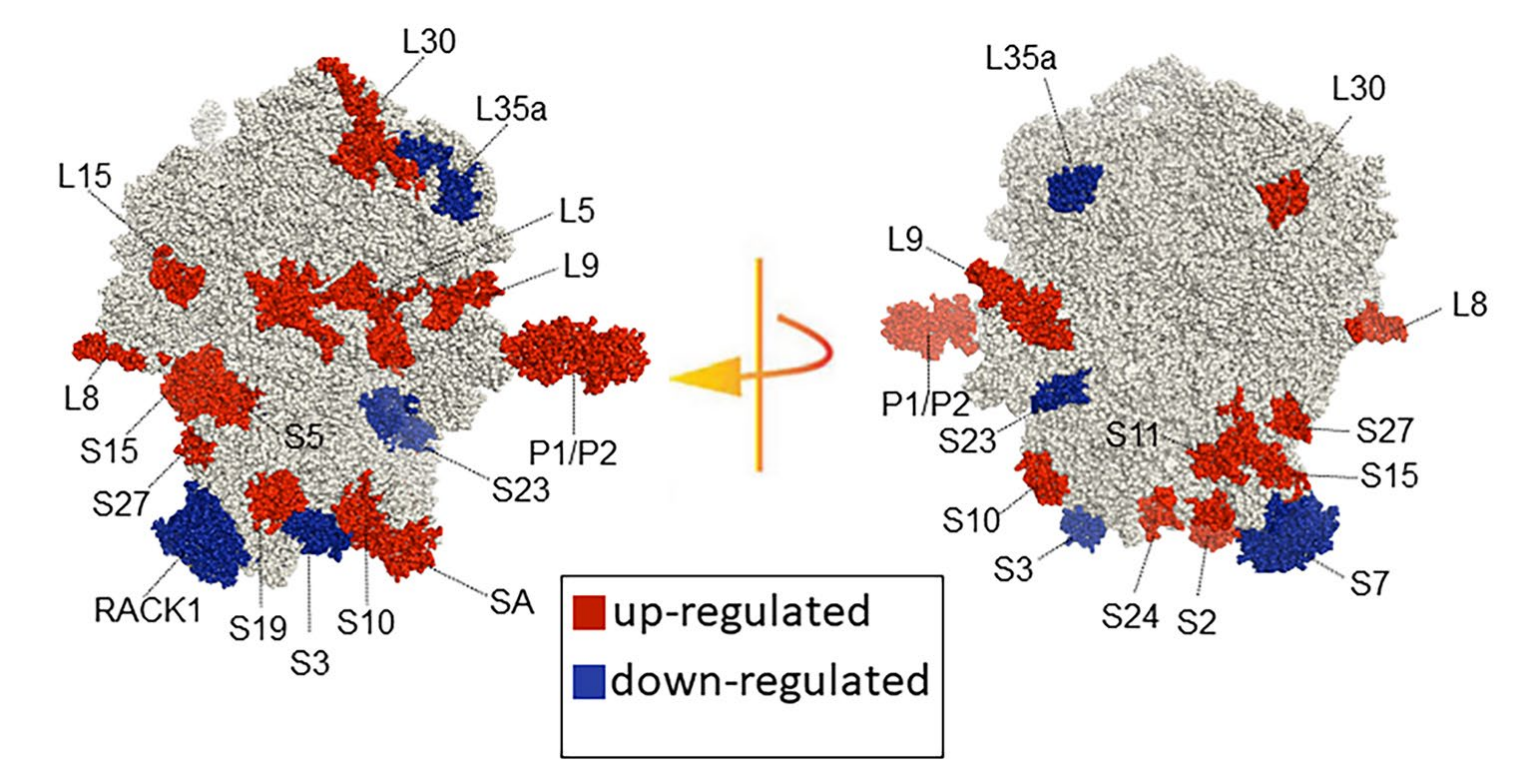
RP remodeling potential of Arabidopsis 80S ribosomes upon INA treatment. The visualization outlines mapped changed protein abundances in response to INA treatment compared to control conditions. Proteomic data were statistically evaluated across individual paralogs within RP families as reported in Supplementary Table S4 and mapped to the wheat 80S monosome used as reference (Armache et al. 2010). Homology of wheat and Arabidopsis RP families was confirmed by protein BLAST matching (Martinez-Seidel et al. 2020a, b). Red and blue represent RP families with increased (red) or decreased (blue) protein abundances of at least one of the RP paralogs. The paralog identities and specific protein changes are reported in Figs. 3 and 4

Relative quantitation proteomic profile analysis showed that 15 proteins of the small subunit were differentially accumulated in response to INA treatment (Fig. 3A). Among them, 4 RPS were downregulated (Fig. 3B) and 11 RPS were upregulated (Fig. 3C). Fold-change of these 15 RPS are also represented as a heat map (Table S7). These results show that the 40S subunit composition changes drastically in response to INA treatment. Interestingly, as can be seen in Fig. 3, among the detected proteins, only one protein per family, and not their paralogs, is affected by INA treatment. Among the 16 deregulated RPS, all the paralogous proteins of families RPSa, RPS2, RPS3, RPS5, RPS24 and RACK1 were detected, whereas not all members were detected for the remaining 9 families. The most strongly deregulated proteins are presented in Fig. 3E. To visualize the spatial distribution and location within the 80S ribosome, the increased and decreased RP families were mapped onto the representation of the 80 S wheat monosome applying different color codes to the significantly changed RP families (Fig. 4). For the mapping, PyMOL visualization software was used to obtain a surface representation and to highlight proteins with significant changes. By choice of rotations, emphasis was given to the proteins that are visible from either the interface- or solvent-sides. These data indicate that specific ribosomal protein paralogs incorporation into ribosomes is differentially regulated in response to INA and suggest that some RPS may play an important role in the stress response.

### Large subunit ribosomal protein levels change in response to INA treatment

We next investigated how INA treatment affects the 60S large subunit proteins (RPL) in the purified samples. Out of the 148 genes encoding large subunit proteins annotated in TAIR10, 80 distinct proteins (Table 2; Table S8) (from 43 families out of a possibility of 48) were found. Members of families RPP3, RPL23a, RPL29, RPL40 and RPL41 were not detected (Tables S3 and S8). When we looked for these proteins in the non-filtered LC-MS/MS data, we found a few RPP3 and RPL23a hits with one unique peptide, but no hits for RPL29, RPL40 nor RPL41. In *Arabidopsis*, RPL41 (a lysine- and arginine-rich 25 amino acids long protein) is currently the only RP that has not been detected by proteomic approaches (Hummel et al., 2015), probably because its tryptic digestion generates peptides that are too small to be detected by LC-MS/MS.

**Table 2:** RPL detected by MS analysis in this study

Family	AGI code	Name	MW (kD)	Razor unique peptides	
***P0***	At3g09200	RPP0B	34.1	15	
	At3g11250	RPP0C	34.4	2	
***P1***	At1g01100	**RPP1A**	11.2	2	
	At5g47700	**RPP1C**	11.2	3	
***P2***	At2g27720	RPP2A	11.4	5	
	At2g27710	**RPP2B**	11.4	9	
***L3***	At1g43170	RPL3A	44.6	33	
	At1g61580	RPL3B	44.5	12	
***L4***	At3g09630	RPL4A	44.7	11	
	At5g02870	RPL4B	44.7	26	
***L5***	At5g39740	**RPL5B**	34.4	17	
***L6***	At1g18540	RPL6A	26.2	24	
	At1g74050	RPL6B	26.0	12	
***L7***	At2g01250	RPL7B	28.1	26	
	At2g44120	RPL7C	28.5	9	
	At3g13580	RPL7D	28.4	5	
***L7a***	At2g47610	RPL7aA	29.1	2	
	At3g62870	RPL7aB	29.0	15	
***L8***	At2g18020	**RPL8A**	27.9	2	
	At4g36130	RPL8C	27.9	15	
***L9***	At1g33120	RPL9A	22.0	16	
	At1g33140	**RPL9B**	22.0	16	
	At4g10450	RPL9D	22.0	5	
***L10***	At1g14320	RPL10A	24.9	3	
	At1g66580	RPL10C	24.1	14	
***L10a***	At1g08360	RPL10aA	24.3	5	
	At2g27530	RPL10aB	24.3	2	
***L11***	At2g42740	RPL11A	20.9	7	
***L12***	At2g37190	RPL12A	18.0	5	
***L13***	At3g49010	RPL13B	18.6	23	
	At5g23900	RPL13C	18.6	10	
***L13a***	At3g07110	RPL13aA	23.5	11	
	At3g24830	RPL13aB	23.5	15	
	At4g13170	RPL13aC	23.6	2	
	At5g48760	RPL13aD	23.6	7	
***L14***	At2g20450	RPL14A	15.5	7	
	At4g27090	RPL14B	15.5	15	
***L15***	At4g16720	**RPL15A**	24.2	12	
***L17***	At1g67430	RPL17B	19.9	10	
***L18***	At3g05590	RPL18B	20.9	22	
	At5g27850	RPL18C	20.9	7	
***L18a***	At1g29965	RPL18aA	21.4	2	
	At2g34480	RPL18aB	21.3	17	
	At3g14600	RPL18aC	21.3	9	
***L19***	At4g16030	RPL19A	24.6	19	
	At1g02780	RPL19B	24.3	8	
	At3g16780	RPL19C	23.3	7	
***L21***	At1g57660	RPL21E	18.7	14	
***L22***	At3g05560	RPL22C	14.0	5	
***L23***	At2g33370	RPL23B	15.0	9	
***L24***	At3g53020	RPL24C	18.6	5	
***L26***	At3g49910	RPL26A	16.9	12	
	At5g67510	RPL26B	16.8	10	
***L27***	At3g22230	RPL27B	15.6	2	
	At4g15000	RPL27C	15.6	4	
***L27a***	At1g70600	RPL27aC	16.5	7	
***L28***	At2g19730	RPL28A	15.9	4	
	At4g29410	RPL28B	15.9	7	
***L30***	At1g36240	RPL30A	12.3	2	
	At3g18740	**RPL30C**	12.3	2	
***L31***	At4g26230	RPL31B	13.8	4	
	At5g56710	RPL31C	13.8	4	
***L32***	At4g18100	RPL32A	15.5	11	
	At5g46430	RPL32B	14.5	2	
***L34***	At1g26880	RPL34A	13.7	4	
	At1g69620	RPL34B	13.7	6	
	At3g28900	RPL34C	13.6	3	
***L35***	At2g39390	RPL35A	14.3	17	
	At3g55170	RPL35C	14.2	3	
	At5g02610	RPL35D	14.3	11	
***L35a***	At1g07070	RPL35aA	12.9	3	
	At1g41880	**RPL35aB**	12.8	6	
***L36***	At5g02450	RPL36C	12.2	9	
***L36a***	At3g23390	RPL36aA	12.1	6	
***L37***	At1g52300	RPL37B	10.8	3	
***L37a***	At3g10950	RPL37aB	10.4	2	
***L38***	At2g43460	RPL38A	8.1	5	
***L39***	At2g25210	RPL39A	6.4	2	

**Fig. 5:**
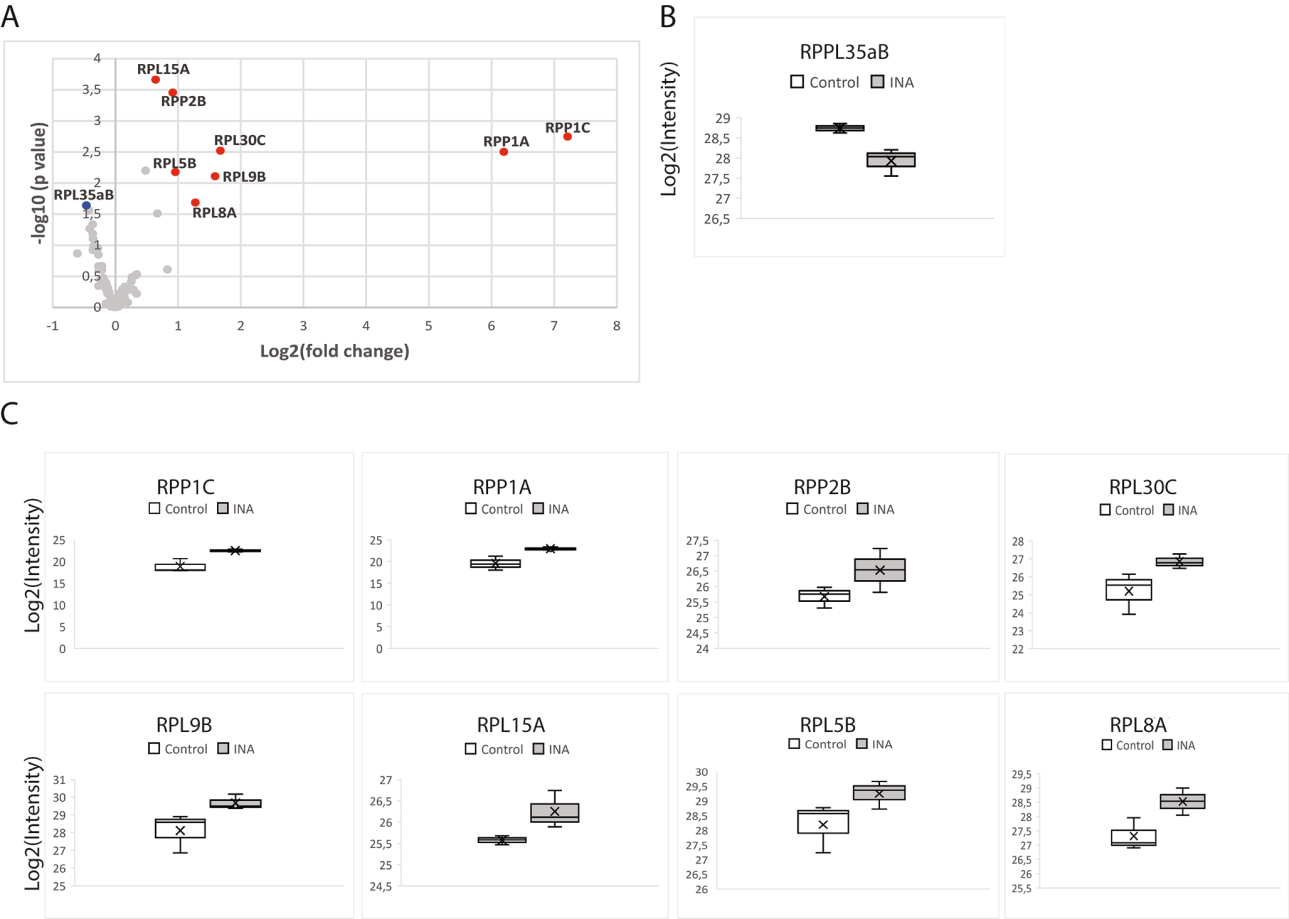
Large subunit ribosomal protein levels change in response to INA treatments Volcano plots of deregulated RPS: downregulated in blue and upregulated in red. Significant deregulation was set as an absolute log_2_ value of fold change ≥ 1.5 and a *q-*values < 0.05 (B, C) Box plots showing the differences and replicate distribution of replicates for each statistically significant downregulated (B) and upregulated (C) proteins. The asterisks represent a significant difference between treated and mock-treated samples. *P-values < 0.05, **P-values < 0.01, limma t-test. (C) Heat map showing the ten most upregulated and downregulated RPS paralogs detected in this study

Using the criteria listed above, a total of 9 RPL were differentially accumulated in the large subunit of ribosomes in response to INA treatment, one protein had reduced levels) Fig. 5B) while 8 had an increased abundance (Fig. 5C), fold-change of these RPL is also represented as a heat map (Table S9). Among these more abundant proteins, induction of RPP1C and RPP1A was more pronounced than the other 6: RPL30C, RPL9B, RPL8A, RPL5B, RPP2B and RPL15A (Fig. 5C). Interestingly, among the detected proteins, only one protein per family is changing in response to INA treatment except for the RPP1 family, for which 2 paralogs are deregulated. These data indicate that the 60S RP composition changes in response to isonicotinic acid treatment. PyMOL visualization software was used to obtain a surface representation and to highlight proteins with significant changes (Fig. 4).

### 5’ upstream analysis of deregulated RPs

**Fig. 6:**
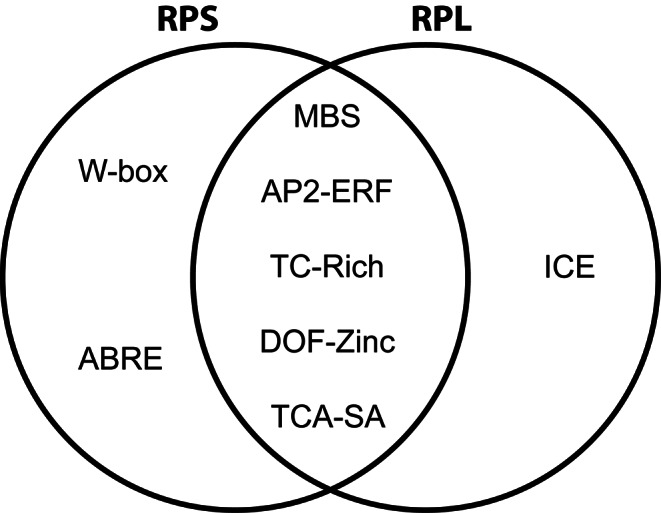
Venn diagram showing of the regulatory elements enriched in the 5’ upstream region of the INA-deregulated ribosomal proteins

Our proteomic study showed that many RPS and RPL are differentially accumulated in the ribosome following INA treatment. To elucidate whether this differential accumulation pattern correlates with the presence of stress or signal-responsive elements in their regulatory regions, we analysed the nucleotide sequences 1 kb upstream of the genes coding for all RP using the PlantCARE and PlantPAN databases. Because several RPs are intronless (Table S2), we only considered the 5’ upstream region for this analysis. This analysis identified multiple stress-responsive elements, distributed in two groups according to their functions: hormone-responsive elements (HREs) and defence/stress-responsive elements (DSREs). Interestingly, both the salicylic acid (SA)-responsive element (TCA-motif) and the abscisic acid (ABA)-responsive elements (ABRE) were enriched in the 5’ upstream regions of the 11 RPS genes showing modified accumulation following INA treatment (*P-*values < 0.05; Supplementary Table S10). In addition, the SA-responsive element was also enriched in the 5’ upstream regions of the 8 RPL genes with increased accumulation following INA treatment (*P-*values < 0.05; Supplementary Table S11). Similarly, all the INA-upregulated RPs have 5’ upstream regions enriched an abiotic responsiveness element (AP2 domain), the dehydration stress responsive element (MBS), a defence and stress-responsive element (TC-rich repeats) and the DOF domain enriched in their (*P-*values < 0.05; Supplementary Table S10, S11). Interestingly, all the RPS with increased abundance showed enrichment of a fungal elicitor-responsive element (W Box) in their 5’ upstream regions (*P-*values < 0.05; Supplementary Table S10). All the stress responsive cis-acting elements are presented in Table S12. Altogether, our analysis demonstrates that a total of seven and six responsive elements are significantly enriched in the 5’ upstream region of the INA regulated RPS and RPL respectively, whilst five of them are common between the RPS and RPL (Fig. 6).

## Discussion

In order to adapt to stress conditions, plants have evolved complex signaling mechanisms implicating various molecular changes to establish appropriate responses. Since protein translation is an energetically demanding process, stress can cause a global drop of protein synthesis (Matsuura et al. 2010; Muñoz and Castellano 2012). However, some proteins are still synthesized during stress to enable cells to tolerate the stress conditions more effectively (Holcik and Sonenberg 2005). In such context, regulation of the identity of some components of the ribosomal subunits may be key to the plant survival under stress conditions (Bailey-Serres et al. 2009; Hummel et al. 2012; Merchante et al. 2017; Solano-De la Cruz et al. 2019). Furthermore, *Arabidopsis* ribosomes are extensively heterogeneous, each individual RP being encoded by two to seven paralogous genes (Weis et al. 2015). With that in mind, it is interesting to speculate that ribosomal protein composition specializes in response to external stimuli to enable the plant adaptation to specific conditions. To address this hypothesis, we immunoprecipitated FLAG-tagged ribosomes followed by protein identification and relative quantitation by LC-MS/MS. This approach allowed us to characterize the abundance of core RP in the *Arabidopsis* ribosome. Our proteomic characterization shows that the majority of RPs encoded by *A. thaliana* are present in the riboproteome. 24 ribosomal proteins (15 RPS and 9 RPL) out of 252 RP-encoding genes showed a significant change in their abundance in response to defense activation. Our results agree with those reported by Hummel et al. (2012), who concluded that different ribosomal protein paralogs are incorporated into the ribosomes depending on growth conditions.

We used untargeted proteomic to address riboproteome (encompassing ribosomal proteins and ribosome-associated proteins) modulation in the context of plant immune activation. Following INA treatment and immunoprecipitation, a total of 1882 non-ribosomal proteins were detected (Table S3). Of those, more than a quarter (508) were only observed following INA treatment, indicating an important rearrangement in ribosome-associated protein following immunity stimulation. These included several proteins with a known link to immunity such as VASCULAR ASSOCIATED DEATH-1 (Lorrain et al. 2004), HSP90 (Huang et al. 2014), PR5 (Zeidler et al. 2004), IMP-α (Palma et al. 2005), BIG and CCT2 (Meteignier et al. 2017). As translational activity and regulation are not solely accomplished by ribosomal protein and require a plethora of accessory proteins, these may represent elements required to fine tune translation in response to stresses.

In eukaryotes, the small subunit of the ribosome makes first contact with the mRNA prior to assembly with the large ribosomal subunit to constitute a translation-competent ribosome. As such, the small subunit is involved in the selection of the mRNAs to be translated and the identity of the ribosomal proteins within the small subunit could impact the identity of the recruited mRNAs. In addition to their crucial roles in translation, specific ribosomal proteins of the small subunit (RPS) are known to play vital roles in abiotic stress and plant-pathogen interactions. In the present study, 11 RPS had increased abundance following the INA treatment (RPS15A, RPS10C, RPS11C, RPS24A, RPS5A, RPS27D, RPS17D, RPS19C, RPS9C, RPS2C and RPSaA, Fig. 3). For some of these RPS, variation in their mRNA levels in response to external stimuli has been previously reported in several plant species. Indeed, the transcript levels of *RPS15A*, the most deregulated RPS in our data, increased significantly in *Arabidopsis* in response to oxidative stress (Saha et al. 2017). Similarly, in the transcriptome of vanilla infected with *Fusarium oxysporum* f. sp. *vanillae*, differential expression of *RPSaA, RPS5A, RPS17D* and *RPS24A* was observed (Solano-De la Cruz et al. 2019), these four RPS are also being upregulated in our riboproteome. In addition, it has been documented that RPS are induced in response to stress in *Oryza sativa*. *RPS9C* and *19C* are among the early responsive genes upregulated under salt stress (Kawasaki et al. 2001), both were also increased in our data. The transcript of some RPS genes accumulated at remarkably high levels (≥ 100 fold) under drought stress (*RPS9C, RPS17D, RPS19C, RPS27D*) or under oxidative stress (*RPS9C*) (Saha et al. 2017); all of these RPS accumulated in our dataset. RPS gene expression was also studied in response to biotic stress in rice. *Xanthomonas oryzae* pv. *oryzae* and *Rhizoctonia solani*, pathogens that respectively cause very serious Bacterial Leaf Blight and Sheath Blight diseases in rice, induced the upregulation of *RPS10C* (29 fold), *RPS9C* (18 fold), and *RPS5A* (14 fold) (Saha et al. 2017), and in our data these same RPS accumulated in response to INA treatment. These reports point toward a differential expression of RPS genes in response to stress treatments leading to a differential accumulation of RPS in the ribosomal apparatus, which might help subunit remodeling and selective translation to cope up with unfavorable conditions.

Interestingly, disease and stress resistance functions of RPL have been reported in recent years. Silencing of *RPL12, RPL19, RPL30* and *RPL10* in *Nicotiana benthamiana* or *Arabidopsis thaliana* compromised nonhost disease resistance against multiple bacterial pathogens (Nagaraj et al. 2015; Ramu et al. 2020); of those, only RPL30C was deregulated in our experiment. In the present study, 8 RPL showed increased abundance following INA treatment. Figure 5 shows the resulting changes in RPP1C, RPP1A, RPL30C, RPL9B, RPL8A, RPL5B, RPP2B and RPL15A. Several reports focused on stress induced differential expression of these RPL. Induction of *RPL30C* with *60S* acidic RP was reported in vanilla infected by *Fusarium oxysporum* f. sp. *vanillae* (Solano-De la Cruz et al. 2019). Similarly, the transcript levels of *RPL30C* increased significantly in response to phytohormones in *Arabidopsis* and in response to oxidative stress in rice (Cherepneva et al. 2003). In our data, we observed a 2,7 fold increased accumulation of RPL30C. In rice, under MeJa and SA treatments, *RPL8A* showed upregulation up to 100 fold, whereas we observed a 2-fold upregulation of RPL8A. Microarray of rice response to *Xanthomonas oryzae* pv. o*ryzae* revealed that *RPL15* was up-regulated more than 10 fold (Moin et al. 2016), while we observed a 1,6-fold increased accumulation of RPL15A.

The enrichment in common *cis-*regulatory elements in the 5’ upstream region of RPS and RPL genes (TCA-motifs, AP2 domain, MBS, TC-rich repeats, and DOF domain) suggests that variation in accumulation is the result of transcriptional changes. As discussed above, the observed changes in riboproteome composition can mostly be explained by higher mRNA levels leading to higher accumulation of ribosomal protein.

While there is evidence of ribosomes with varying composition, there is little understanding of how the cell regulates ribosome heterogeneity. It can occur in part during ribosome biogenesis, a complex process taking place in the nucleolus and involving association of ribosomal proteins with rRNA to constitute the ribosomal subunits. Previous studies have measured differential ribosomal protein levels in the nucleus following immunity elicitation (Ayash et al. 2021; Bae et al. 2003; Fakih et al. 2016; Howden et al. 2017). We previously reported that some ribosomal proteins were overrepresented in the nucleus after chitosan elicitor treatment (RPSaA, RPS5A, RPS9C, RPS10C, RPS11C, RPS17D, RPS19C, RPS24A, RPS27D and RPL30C) (Fakih et al. 2016). RPS5A and RPS11C were also found to have a significant change in their abundance in the Arabidopsis nucleus during PTI (Ayash et al. 2021). In tomato (*Solanum lycopersicum*), five RPS (RPSaA, RPS5A, RPS10C, RPS17D and RPS19C) were more abundant in the nucleus during infection by the oomycete pathogen *Phytophthora capsici* (Howden et al. 2017). These reports support a probable role of RPs during the plant immune response. Additionally, switching between RPs which are assembled onto the pre-ribosomal subunits in the nucleolus is possible before the new ribosomes are functional (Genuth and Barna 2018).

In summary, the findings of this study open new and interesting avenues for research in ribosome composition during biotic and abiotic stress. The mass spectrometry approach used here detected a total of 52 RPS and 80 RPL of which 15 and 9 were deregulated respectively. However, a number of ribosomal proteins were undetected. It is possible that they were undetected because they were at an abundance level below the detection threshold of the mass spectrometer or because of technical reason, we cannot exclude that these undetected proteins were not deregulated. In addition, one limitation of the approach used in our study is that it is not possible to state if the co-IPs in both treatments have the same amount of free versus translating ribosomes. While the conclusions drawn for the RPL could be affected by this fact, the ones for the RPS should not, since if RPS can be immunoprecipitated using RPL18-FLAG these have to be from assembled ribosomes. Our study expands our molecular knowledge of the ribosomal proteins and ribosome-associated proteins and highlights the importance of studying the function of individual RPs paralogs by genetic analysis of ribosomal protein mutants to clarify their roles in response to stresses. Future work will be aimed at unraveling the specific mechanisms by which RPs affects the plant defense.

## Electronic supplementary material

Below is the link to the electronic supplementary material.

Supplementary Material 1

Supplementary Material 2

## Data Availability

All raw proteomic data is available at: MassiVE repository (https://massive.ucsd.edu/ProteoSAFe/static/massive.jsp) with the identifier MSV000089714.
