# Supplementary figures and images for: Specific alterations in riboproteomes composition of isonicotinic acid treated arabidopsis seedlings
Source: Plant Mol Biol. 2023 Feb 15;111(4-5):379–92. doi: 10.1007/s11103-022-01332-2 (PMC10090002; doi:10.1007/s11103-022-01332-2)

**Supplementary Figure 1:**


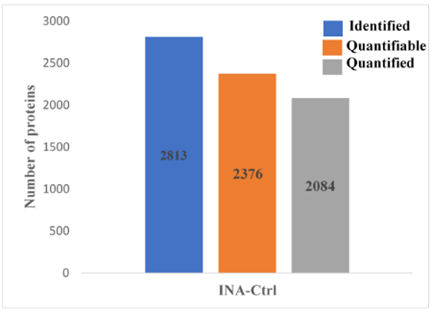


**Figure S2**


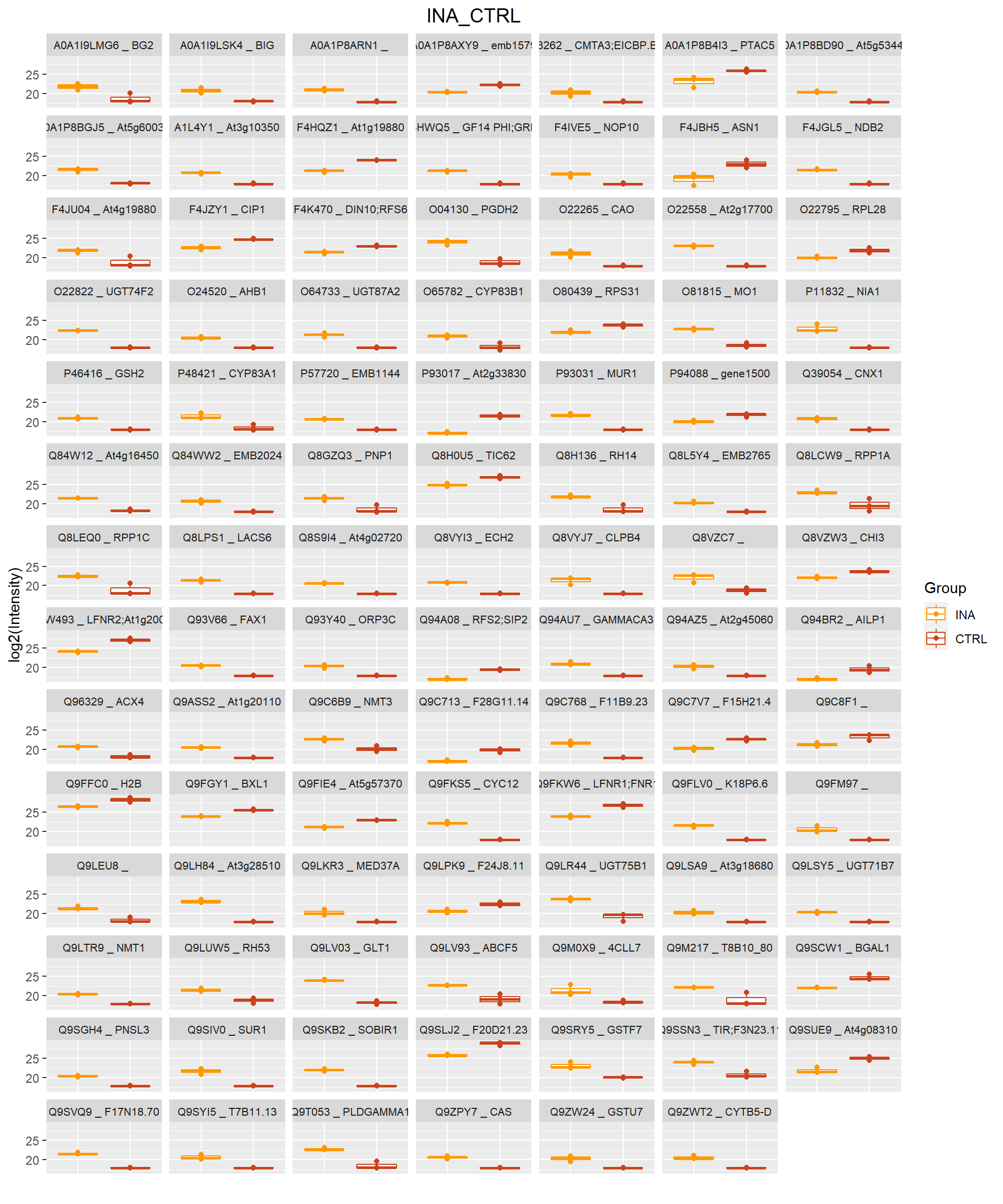

Supplement: Supplementary file 1 — Supplementary Material 1 [file 11103_2022_1332_MOESM1_ESM.docx]
